# The role of detailed medical history for the early diagnosis of familial bradycardia in a patient with associated atrial fibrillation: case report

**DOI:** 10.1093/ehjcr/ytae116

**Published:** 2024-02-28

**Authors:** Andreea Ciacaru, Anna Tusa, Annamaria Magdas, Cristian Podoleanu

**Affiliations:** Cardiology Department, Mures County Hospital, Targu Mures, Romania; Internal Medicine Department 3, Mures County Hospital, Targu Mures, Romania; Internal Medicine Department, George Emil Palade University of Medicine, Pharmacy, Science, and Technology, Targu Mures, Romania; Cardiology Department, George Emil Palade University of Medicine, Pharmacy, Science, and Technology, Targu Mures, Romania

**Keywords:** Familial bradycardia, LMNA mutation, Syncope, Case report

## Abstract

**Background:**

Bradycardia represents a frequent reason for medical presentation and has a complex aetiology, including genetic disorders, like LMNA mutation. LMNA mutation is responsible for laminopathies, including LMNA -cardiomyopathy. Cardiac involvement is prevalent and is linked to dilated cardiomyopathy associated with conduction block, which is anticipated by bradyarrhythmia and supraventricular tachyarrhythmia. LMNA mutation carriers have higher risk for sudden cardiac death (SCD), malignant ventricular tachycardia, and extreme bradycardia.

**Case summary:**

A 48-year-old female presented for recurrent episodes of dizziness, lightheadedness, headache, and fatigue, occurring at rest. The past medical history was positive for hypertension and one episode of paroxysmal atrial fibrillation. The family medical history was positive; both children and the patient’s mother are known with bradycardia. The electrocardiogram showed sinus bradycardia, and the echocardiography revealed a mild concentric hypertrophy of the left ventricle, associated with impaired relaxation diastolic dysfunction. The 24 h Holter monitoring recorded sinus bradycardia, multiple pauses, paroxysmal atrial fibrillation, and multiple episodes of junctional rhythm. The positive family medical history suggested a genetic link. Further, genetic testing was performed, revealing a mutation of the LMNA gene.

**Discussion:**

Despite apparently benign at the initial presentation, the correct diagnosis and management required detailed medical history and extensive investigation of both the patient and the first-degree relatives. As the LMNA mutation carriers have a higher risk for SCD and have a mortality risk of 40% at 5 years, we emphasize the role of early diagnosis and periodic monitoring for preventing the worsening of the condition.

**Learning points**

- LMNA mutation carriers have a greater risk for sudden cardiac death, malignant ventricular tachycardia, and extreme bradycardia.
- Detailed medical history and extensive investigation of the patient and of the first-degree relatives is required for early diagnostic of familial bradycardia and further management.

## Introduction

The LMNA gene is located on the chromosome 1q21.1-21.2, and it encodes the nuclear membrane protein lamin A/C. This protein plays an important role in nuclear stability and functions, regulating gene transcription through modulation of chromatin organization, DNA replication, and signal transduction pathways. LMNA mutation can be inherited in an autosomal dominant manner and is responsible for a group of diseases called laminopathies which may involve different organs, including the heart (cardiolaminopathy).^1^ Typically, the initial pathophysiological abnormalities of the cardiolaminopathy include rhythm disorders like bradyarrhythmia and supraventricular tachyarrhythmias and are followed by dilated cardiomyopathy (DCM) in the later stages of the disease.^1^

## Summary figure

**Figure:**
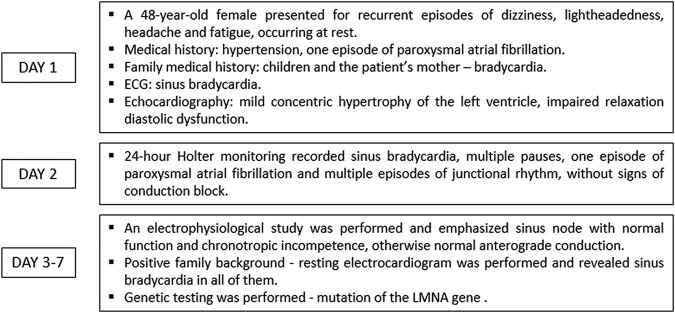


## Case presentation

**Figure 1:**
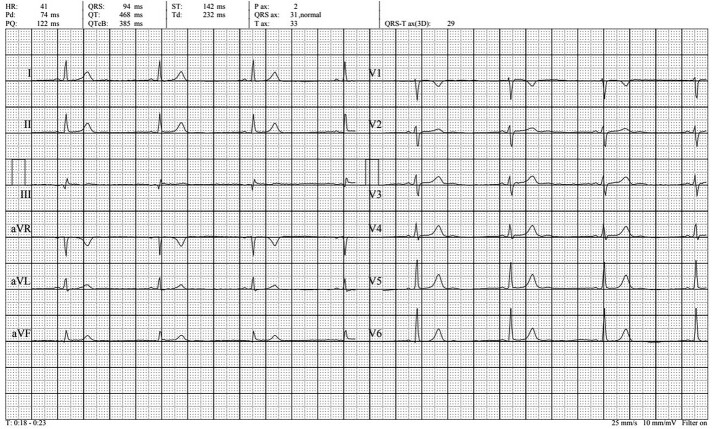
Resting 12-lead electrocardiogram of the patient showing sinus bradycardia.

**Figure 2:**
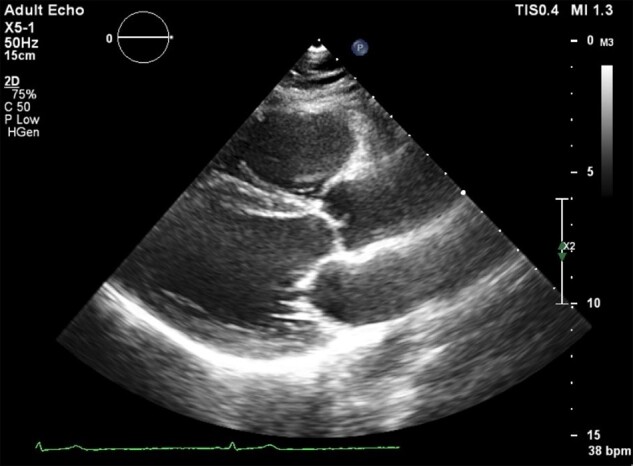
Echocardiography section from parasternal long-axis view showing a mild concentric hypertrophy of the left ventricle.

**Figure 3:**
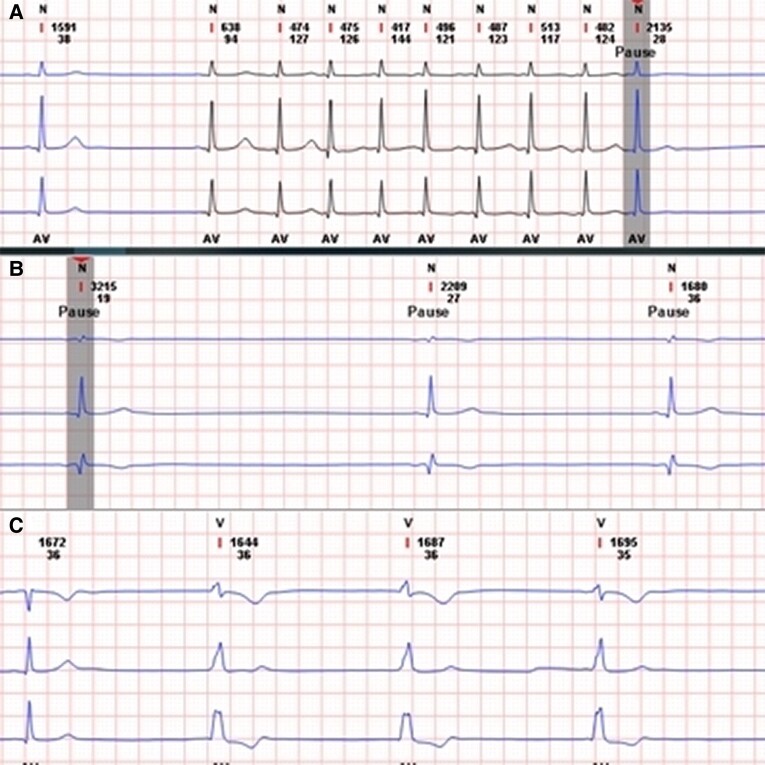
Twenty-four hour Holter monitoring: (*A*) paroxysmal atrial fibrillation; (*B*) the longest recorded pause of 3215 ms, during the night (12:41 a.m.); and (*C*) junctional rhythm.

**Figure 4:**
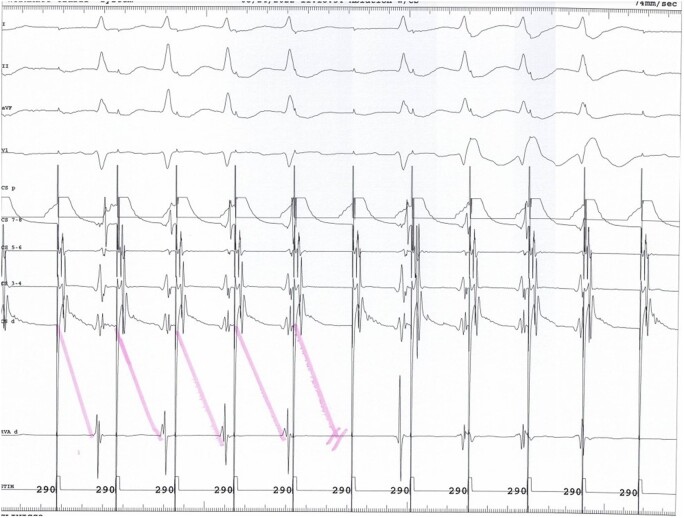
Electrophysiological study—inducing anterograde Wenckebach periodicity.

**Figure 5:**
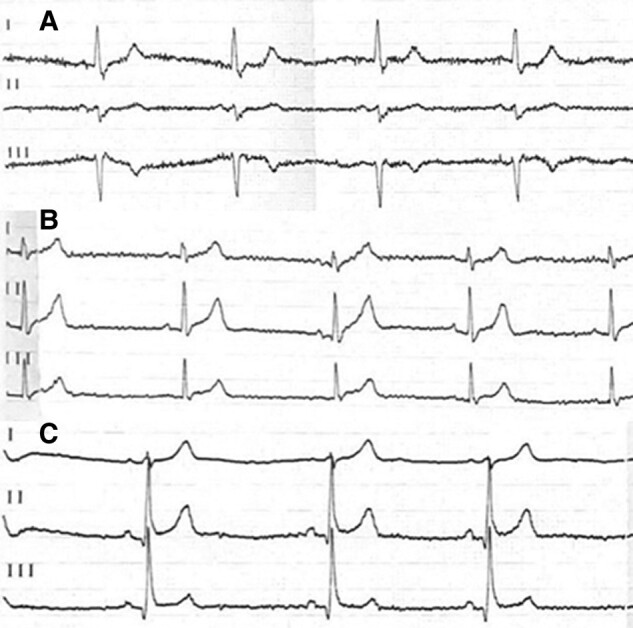
Resting 12-lead electrocardiogram of the first-degree relatives: (*A*) electrocardiogram of the patient’s mother. (*B*) The 20-year-old first son. (*C*) The 16-year-old second son.

A 48-year-old female presented to the department of cardiology with recurrent episodes of dizziness, lightheadedness, headache, and fatigue occurring at rest, occasionally associated with elevated arterial blood pressure up to 180/90 mmHg. The past medical history was suggestive for arterial hypertension, sub-optimally treated with perindopril 5 mg once daily, and one episode of paroxysmal atrial fibrillation occurring when the patient was 15 years old. Detailed medical history revealed cardiovascular conditions of the first-degree relatives as well: the mother of the patient was diagnosed with bradycardia and high blood pressure and the two male children of the patient (aged 16 and 20 years, respectively) were also diagnosed with asymptomatic bradycardia. During the physical exam, we measured a heart rate of 44 b.p.m. at rest, but no other significant findings were noted. The resting electrocardiogram recorded sinus rhythm, with a heart rate of 41 b.p.m, QRS axis at 31 degrees, PQ interval of 122 ms, QRS complex of 94 ms, and negative T-wave in V1 (*Figure 1*). The echocardiography revealed mild concentric hypertrophy of the left ventricle, associated with impaired relaxation diastolic dysfunction (*Figure 2*). A 24 h Holter monitoring was performed, which recorded more than 30 000 pauses longer than 1500 ms (32 187 pauses), with a maximum length of 3215 ms, during the night (12:41 a.m.). The medium heart rate was 41 b.p.m., while the minimum and the maximum was 25 b.p.m. and 93 b.p.m., respectively. A paroxysmal episode of atrial fibrillation with a duration of 13 min was recorded, as well as multiple episodes of junctional rhythm, but no atrioventricular (AV) conduction disorders (*Figure 3*). Supine and upright blood pressure measurements were suggestive for orthostatic hypotension, and head-up tilt table test was performed. During the passive phase of the tilt testing, a clinically significant decrease in the blood pressure was recorded, associated with a transient loss of consciousness at a minimal heart rate of 46 b.p.m. at the onset of the symptoms. An electrophysiological study was performed and emphasized sinus node with normal function and chronotropic incompetence but normal anterograde conduction (*Figure 4*). Taking into consideration the positive family history for bradycardia, a resting electrocardiogram was performed in all the first-degree relatives, and it showed sinus bradycardia in all of them (*Figure 5*). The echocardiography of the children did not show signs of structural cardiac disease, while the mother of the patient had a mild aortic and mitral regurgitation, with mild hypertrophy of the left ventricle. The patient and her sons underwent sequential genetic testing which revealed a mutation of the LMNA gene, pathogenic variant c.686T>C (p.Ile229Thr) for the patient, but both children were negative for the mutation. The differential diagnosis with myocarditis or infiltrative myocardial disease was addressed by performing cardiac magnetic resonance imaging which did not show any abnormality suggestive for the abovementioned conditions.

The patient was discharged on treatment with perindopril, midodrine, and dabigatran. At 6-month follow-up, a 24 h Holter ECG monitoring was performed, recording an average heart rate of 42 b.p.m., with a minimum heart rate of 29 b.p.m. during the night and a maximum heart rate of 78 b.p.m. The monitoring recorded 24 959 pause intervals longer than 1.5 s, and the longest pause had 2.887 s at 02:14 am. The monitoring did not detect any signs of atrial fibrillation or conduction disorders. The echocardiography showed normal systolic function, without signs of dilatated cardiomyopathy or other structural signs of disease.

## Discussion

The bradycardic rhythm disorders have a broad aetiology (infiltrative diseases, electrolyte imbalance, neurological or metabolic disorders, and many others) and are frequently seen in the cardiological practice, occasionally without being associated with a clinically manifest heart disease.^2^ Intrinsic causes include genetic disorders due to gene mutations associated with bradyarrhythmia, like mutations of the HCN4, SCN5A, LMNA, and other genes.^3^ The LMNA gene mutation is the second most common gene mutation associated with familial cardiomyopathy, and it was identified in one-third of the cases presenting with DCM and conduction disorders.^4^ LMNA gene mutation carriers have a mortality risk of 40% at 5 years due to increased risk for cardiac events such as sudden cardiac death (SCD), malignant ventricular tachycardia (VT), extreme bradycardia due to high-degree AV block (AVB), and severe heart failure.^1^ The clinical presentation is elusive: in the early stages of LMNA cardiomyopathy, the only abnormalities described are bradycardic rhythm disorders or conduction defects of variable degree and/or elevated level of high-sensitivity troponin T follower, while dilatative cardiomyopathy is present in the later stages of the disease.^1,5^ In some cases, cardiac magnetic resonance investigation describes a typical midmyocardial septal late-gadolinium enhancement in LMNA mutation carriers.^6^ To our best knowledge, the current management of these patients relies on the early risk assessment by the risk prediction score for life-threatening ventricular tachyarrhythmias in laminopathies at 5 years, which includes following high-risk factors: male sex, non-missense LMNA mutation, presence of AVB and/or non-sustained VT, and left ventricular ejection fraction < 45%.^7^ Also, according to the guidelines, in patients with DCM/hypokinetic non-dilated cardiomyopathy (HNDCM) and a 5-year estimated risk prediction score ≥ 10% associated with non-sustained VT, left ventricle ejection fraction < 50%, or AV conduction delays, there is a class IIa indication for ICD implantation for primary prevention.^7^

With our case report, we emphasize the importance of detailed medical history and extensive investigation of the patient and his relatives of first degree. Given the actual level of knowledge on the long-term management of such patients, early diagnosis and periodic monitoring to prevent the worsening of the condition is of utmost importance.

## Data Availability

The data underlying this article will be shared on reasonable request to the corresponding author.

## Lead author biography

Andreea Ciacaru is a cardiology resident at the Cardiology Department, Mures County Hospital, Targu Mures, Romania. She obtained her medical degree at the “George Emil Palade” University of Medicine, Pharmacy, Science, and Technology, Targu Mures.
